# Miniaturized QuEChERS extraction method for the detection of multi-residue pesticides in bat muscle tissue

**DOI:** 10.1038/s41598-022-11352-z

**Published:** 2022-05-03

**Authors:** Camila Guimarães Torquetti, Mirna Maciel d’Auriol-Souza, Leiliane Coelho André, Ana Tereza Bittencourt Guimarães, Benito Soto-Blanco

**Affiliations:** 1 Departamento de Clínica E Cirurgia Veterinárias, Escola de Veterinária, Universidade Federal de Minas Gerais (UFMG), Belo Horizonte, MG Brazil; 2 Departamento de Análises Clínicas E Toxicológicas, Faculdade de Farmácia, Universidade Federal de Minas Gerais (UFMG), Belo Horizonte, MG Brazil; 3 Laboratório de Investigações Biológicas, Centro de Ciências Biológicas E da Saúde, Universidade Estadual Do Oeste Do Paraná (Unioeste), Cascavel, PR Brazil

**Keywords:** Environmental sciences, Environmental chemistry

## Abstract

Habitat loss and fragmentation are among the greatest threats to biodiversity and ecosystem stability, with physiological implications on wild fauna. Bats (Microchiroptera) are small mammals with a wide variety of eating habits, and the well-being of these animals is disturbed by exposure to pesticides. This study aimed to develop a miniaturized QuEChERS (Quick, Easy, Cheap, Effective, Rugged, and Safe) extraction method for the detection of multi-residue pesticides in bat muscle tissue using gas chromatography coupled with mass spectrometry (GC–MS). A total of 48 pesticides were tested in 250 mg of bat muscle tissue. The developed analytical method was applied to 148 bats collected from two different areas in Minas Gerais State, Southeast Region of Brazil. The method presented good sensitivity and allowed the determination of residues of 48 pesticides in bat muscle using GC–MS. The miniaturized extraction method makes the analysis feasible even when the sample volume is limited. However, no pesticide residues were detected in bats from the two areas investigated.

## Introduction

Environmental contamination by pesticides exerts both direct and indirect impacts on ecosystems^1,2^. These impacts include a reduction in biodiversity^3,4^ and a decline in the population of several species, including bats^2,5–7^, birds^8^, and amphibians^9,10^. The determination of environmental contamination by pesticides can provide a toxicological risk assessment of the evaluated species. The exposure of animals to pesticides can be assessed by determining residual pesticide levels in tissues, usually performed via gas chromatography coupled with mass spectrometry (GC–MS), which allows the separation and detection of a mixture of components with high analytical sensitivity^11,12^. Because of the complex nature of the samples and the low concentrations of pesticides present in animals with small body mass, it is crucial to extract and concentrate the analytes of interest during sample preparation while removing possible interferents^13^.

The Quick, Easy, Cheap, Effective, Rugged, and Safe (QuEChERS) extraction method was developed as a simple multi-residue method that can be performed in any laboratory, without the need for sophisticated equipment^14^. This method was initially proposed for the extraction of pesticide residues from vegetable matrices; however, owing to its simplicity and efficacy, it has been adapted and optimized for use in other types of matrices, including animal tissues^15,16^, milk^17^, honey^18–20^, water^21,22^, and soil^23,24^.

The original QuEChERS method requires 10 g of sample^14^, which is not always available for smaller sample sizes. Therefore, miniaturization of QuEChERS is an alternative technique for analyzing small samples^25–27^. In addition, the miniaturized method uses fewer reagents and solvents, is relatively cheaper, and reduces environmental impact compared to traditional methods^28^.

Bats (Microchiroptera) are small mammals with a wide variety of eating habits; thus, they play an important ecosystem service in maintaining biomes through seed dispersal, pollination, and the control of insect and small vertebrate populations^29^. The first reports on bat mortality from pesticides were published in the early 1950s^30,31^. Other studies have reported on the exposure of bats to pesticides, primarily organochlorines, via the determination of residues and their effects, as well as the determination of lethal doses and concentrations of the pesticides^2^. Recently, there has been an increased interest in evaluating the effects of prolonged exposure to pesticides^2^ on living organisms. However, assessments of natural populations remain scarce^32,33^.

The determination of pesticide residues in most bat species is challenging because of their small body masses: individual animals can weigh less than 10 g^34^. Therefore, this study aimed to develop a miniaturized QuEChERS extraction method for detecting multi-residue pesticides in bat muscle tissue using GC–MS. The developed method uses fewer reagents and less bat tissue than traditional techniques.

## Materials and methods

### Standards and reagents

Analytical-grade reagents for high performance liquid chromatography (HPLC) analysis, including acetonitrile (J. T. Baker, Mexico), ethyl acetate (J. T. Baker, Mexico), hexane (Merck, Darmstadt, Germany), primary and secondary amines (PSA; Agilent, USA), octadecylsilane (C18; Agilent, Santa Clara, CA, USA), magnesium sulfate (St. Louis, MO, USA), and acetone (Scharlau, Barcelona, Spain), were used in this study. Ultrapure water was obtained using a Millipore Q UV3 purification system (Merck, Milford, CT, USA). Analytical standards of investigated pesticides were provided (> 98.0% purity grade) by Dr. Ehrenstorfer (Augsburg, Germany) and AccuStandard (New Haven, CT, USA).

### Animals

The experimental design and animal collection were approved by the Ethics Committee on the Use of Animals at the Federal University of Minas Gerais (Protocol CEUA 166/2017) and Chico Mendes Institute for Conservation and Biodiversity (Protocol ICMBio 57,026-1).

Two areas with different anthropic pressures were chosen for bat collection: one in a rural area of the Uberaba municipality, MG, Brazil (19°45′43'' S' and 48°06′05'' W), characterized by intense agricultural activity^35^, and the other in the National Park (PARNA) of Serra do Cipó, Santana do Riacho, MG, Brazil, a Brazilian federal conservation unit^36^. The bats were collected in 2018 and 2019 using 10–12 m long mist nets, which were opened at dusk on trails, fragments of forest, and in the vicinity of day shelters. The mist nets remained open for approximately 4 h (18:00–22:00 h) and were inspected at intervals of 20–30 min. Capture procedures were conducted in conformity with the American Society of Mammalogists^37^. A total of 148 bats were collected: 78 from the agricultural region of Uberaba and 70 from the PARNA federal conservation unit. The animals were placed in individual cloth bags until euthanasia was performed. The animals were then placed in a plastic bag containing a cotton pad, which was previously immersed in isoflurane, to induce loss of consciousness, followed by an intraperitoneal injection of an anesthetic (ketamine hydrochloride). The bats were then stored in a freezer at − 20 °C until analysis.

### Optimization of sample extraction and cleanup

The choice of tissues for the chromatographic analysis was based on previous studies, which indicated that higher concentrations of pesticide and other xenobiotic residues can be found in the liver, fat, and muscle tissues^38–40^. Consequently, because bats have little fat, muscle was used as a matrix due to its large abundance^41^. However, because the liver was insufficient for analysis, especially in smaller species, fragments of fat and liver from larger bats were collected to perform a comparative analysis between different types of tissues.

Two extraction methods, using 1.0 g (method A) and 250 mg (method B) of bat muscle tissue, were compared.

Method A is based on a modified QuEChERS extraction method described by Oliveira et al.^15^. Water (3.6 mL), acetonitrile (5.0 mL), and ethyl acetate (2.14 mL) were added to 1.0 g of sample, and the mixture was vortexed for 1 min at 2200 rpm. This was followed by the addition of MgSO_4_ (2.86 mg) and sodium acetate (0.71 mg), which were then homogenized in a vortex for 1 min at 2200 rpm and centrifuged for 11 min at 4000 rpm. The samples were then kept at − 20 °C overnight. Next, the samples were centrifuged for 5 min at 4000 rpm, and the extract (1.0 mL) was subsequently transferred to a microcentrifuge tube containing MgSO_4_ (150 mg), PSA (30 mg), and C18 (30 mg). After stirring at room temperature (for 1 min at 2200 rpm) and centrifugation (for 12 min at 9000 rpm), the supernatant was injected into the GC–MS instrument.

Method B is based on the miniaturized QuEChERS extraction method proposed by Brandhonneur et al.^25^. The samples were thawed and fragments of the pectoral muscle (250 mg) were removed, dehydrated, and homogenized with MgSO_4_ (400 mg). Acetonitrile (1.4 mL), hexane (200 µL), and azoxystrobin (1.2 ng/mL, for process control) were added to each sample. The samples were vortexed for 5 min at 2200 rpm and placed in a freezer at − 20 °C for 30 min. The samples were then centrifuged for 20 min at 5000 rpm. Next, the organic phase (800 µL) was transferred to a microcentrifuge tube containing MgSO_4_ (100 mg), PSA (50 mg), and C18 (50 mg). After vortexing for 1 min at 2200 rpm, the samples were placed on a shaker for 10 min at room temperature and then centrifuged for 12 min at 12,000 rpm at 10 °C. The organic phase (150 µL) was transferred into a vial equipped with an insert to evaporate the solvent at room temperature. The samples were reconstituted with acetone (75 µL), vortexed for 30 s at 2200 rpm, and the solution (8 µL) was then injected into the GC–MS instrument.

Azoxystrobin (batch standard G128076 from Dr. Ehrenstorfer, Germany) in acetonitrile (1.2 ng/mL) was used as the process control. All samples, including white samples (non-spiked samples), were fortified with 440 µL of azoxystrobin (1.2 ng/mL). The extraction was considered satisfactory when the azoxystrobin recovery rate varied between 80 and 110%^42^.

After determining the best extraction method (A or B), the bat muscle fragment was fortified with a pesticide stock solution and extracted to determine the retention time (RT) and ions for the selected ion monitoring (SIM) mode chromatography.

### Chromatographic system

Chromatographic analyses were performed using a GC–MS instrument (Agilent 7890A-5975C) equipped with an automatic sampler (Agilent Sampler 80). Chromatographic separation was performed using a capillary column DB-5 (30 m × 0.25 mm × 0.25 µm; Agilent Technologies, USA) with He (99.999%; Air Products, Brazil) as the carrier gas at a flow rate of 1.2 mL/min. The chromatographic conditions included an injector temperature of 250 °C, injection volume of 8 µL in splitless mode, a column temperature ramp from 60 to 160 °C with three heating rate ramps of 20 °C/min, followed by an increase to 255 °C at 5 °C/min, and then a ramp of 20 °C/min up to a final temperature of 280 °C, which was maintained for 7 min. The post-run time was 2 min at 280 °C, with a He flow rate of 2.6 mL/min. The total chromatographic runtime was 32.25 min. The injection syringe was washed three times with acetone–water (1:1 v/v) and acetonitrile between the injections. The spectrometer was set at an impact ionization voltage of 70 eV, ionization source temperature of 230 °C, quadrupole temperature of 150 °C, and interface temperature of 300 °C.

The software used for data acquisition was the MSD ChemStation. Data acquisition started at 3.5 min in the full-scan mode, with a mass range between 50 and 450 m/z in the SIM mode. The pesticides were confirmed by comparing the results with the data from the National Institute of Standards and Technology (NIST) library database. SIM mode was used for the identification of compounds in standard solutions, and the monitored ions and RTs are listed in Table 1.

**Table 1:** Chemical formula, molecular mass, retention time (RT), and detection ions (m/z) of the compounds analyzed via gas chromatography coupled with mass spectrometry (GC–MS).

Compound	Chemical formula	Molecular mass	RT	Ion 1 (m/z)	Ion 2 (m/z)	Ion 3 (m/z)
Alachlor	C_14_H_20_ClNO_2_	269.77	12.63	269.00	188.00	160.00
Aldrin	C_12_H_8_Cl_6_	364.91	13.95	292.90	262.90	79.00
Azoxystrobin	C_22_H_17_N_3_O_5_	344.00	29.64	403.10	388.10	344.00
Bifenthrin	C_23_H_22_ClF_3_O_2_	422.868	21.98	422.10	181.00	186.00
Bromophos-methyl	C_8_H_8_BrCl_2_O_3_PS	365.996	14.61	330.80	212.80	124.80
Bromopropylate	C_17_H_16_Br_2_O_3_	428.12	21.93	427.80	340.80	182.80
Captan	C_9_H_8_Cl_3_NO_2_S	300.589	15.57	263.80	148.90	78.90
Carbophenothion	C_11_H_16_ClO_2_PS_3_	342.865	19.72	341.90	156.90	96.90
Chlorfenapyr	C_15_H_11_BrClF_3_N_2_O	407.61	17.99	407.90	247.00	58.90
Chlorothalonil	C_8_Cl_4_N_2_	265.911	11.12	265.80	228.90	193.90
Chlorpyrifos-methyl	C_7_H_7_Cl_3_NO_3_PS	320.90	12.38	285.80	124.90	78.90
Chlorthiophos	C_11_H_15_Cl_2_O_3_PS_2_	361.245	18.93	359.90	268.80	96.80
Cyfluthrin	C_22_H_18_Cl_2_FNO_3_	434.288	26.14	433.00	226.00	162.00
Cypermethrin	C_22_H_19_Cl_2_NO_3_	415.07	26.45	315.10	181.00	162.90
DDD 2,4	C_14_H_10_Cl_4_	320.041	17.54	234.90	198.90	165.00
DDE 4,4	C_14_H_8_Cl_4_	318.025	16.18	317.80	245.90	176.00
DDT 2,4	C_14_H_9_Cl_5_	354.486	18.84	353.80	234.80	198.80
Dicofol	C_14_H_9_Cl_5_O	370.486	14.39	249.90	138.90	110.90
Dieldrin	C_12_H_8_Cl_6_O	377.87	17.41	379.80	276.80	251.90
Endosulfan I	C_9_H_6_Cl_6_O_3_S	403.82	16.49	240.80	206.90	194.80
Endosulfan II	C_9_H_6_Cl_6_O_3_S	403.82	18.22	407.70	268.80	170.00
Endosulfan sulfate	C_9_H_6_Cl_6_O_4_S	419.81	19.86	421.80	386.80	236.80
Endrin	C_12_H_8_Cl_6_O	380.91	18.11	379.90	262.80	80.90
Fenarimol	C_17_H_12_Cl_2_N_2_O	330.03	24.02	330.00	218.90	138.90
Fenitrothion	C_9_H_12_NO_5_PS	277.02	13.39	276.90	260.00	124.90
Fenpropathrin	C_22_H_23_NO_3_	349.4229	22.28	349.10	181.00	97.00
Fenvalerate	C_25_H_22_ClNO_3_	419.900	28.05	419.10	167.00	124.90
Folpet	C_9_H_4_Cl_3_NO_2_S	296.558	15.77	294.00	103.90	75.80
HCH alpha	C_6_H_6_Cl_6_	290.83	9.71	353.70	218.80	180.80
HCH beta	C_6_H_6_Cl_6_	290.83	10.42	253.80	218.80	180.80
HCH delta	C_6_H_6_Cl_6_	290.83	11.47	253.70	218.80	180.80
HCH gamma	C_6_Cl_6_	284.782	10.63	253.80	218.80	180.80
Heptachlor	C_10_H_5_Cl_7_	369.82	12.82	371.80	271.80	99.90
Heptacloro epoxid	C_10_H_5_Cl_7_O	389.317	15.25	387.80	352.90	80.90
Lambda cyhalothrin	C_23_H_19_ClF_3_NO_3_	449.10	23.89	449.10	209.00	181.00
Methoxychlor	C16H15Cl3O2	344.01	22.12	344.00	227.00	152.00
Mirex	C_10_Cl_12_	539.63	23.60	331.70	271.60	236.70
Ovex (Clorfenson)	C_12_H_8_Cl_2_O_3_S	303.161	16.93	301.90	174.90	110.90
Oxyfluorfen	C_15_H_11_ClF_3_NO_4_	361.700	17.67	361.00	299.90	252.00
Parathion-methyl	C_8_H_10_NO_5_PS	263.00	12.59	262.90	124.90	108.90
Permethrin	C_21_H_20_Cl_2_O_3_	390.08	25.27	207.00	183.00	162.90
Phosalone	C_12_H_15_ClNO_4_PS_2_	366.99	23.02	366.90	181.90	120.90
Procymidone	C_14_H_11_Cl_2_NO_2_	296.149	15.69	282.90	254.90	96.00
Profenofos	C_11_H_15_BrClO_3_PS	371.94	17.23	373.90	338.90	138.90
Prothiofos	C_11_H_15_Cl_2_O_2_PS_2_	345.245	17.04	308.90	266.90	112.80
Quintozene	C_6_Cl_5_NO_2_	295.335	10.48	294.80	264.60	236.70
Tetradifon	C_12_H_6_Cl_14_O_2_S	353.88	22.81	355.80	239.10	98.00
Trifluralin	C_13_H_16_F_3_N_3_O_4_	335.2790	9.15	306.00	290.00	263.90
Vinclozolin	C_12_H_9_Cl_2_NO_3_	286.111	12.52	284.90	211.90	197.90

### Optimization of chromatographic conditions

A standard stock solution containing 69 pesticides was used. One thousand microliters of the stock solution in acetonitrile-ethyl acetate (7:3 v/v) was injected into the GC–MS instrument. The working solutions of each pesticide are listed in Table 2.

**Table 2:** Retention time (RT), stock and working solutions, recovery and probability obtained from the NIST library of thecompounds analyzed via gas chromatography coupled with mass spectrometry (GC–MS).

Pesticide	RT	Stock solution (ng/µL)	Working solution (ng/µL)	Recovery	Probability (NIST)
				40–120%	%
Alachlor	12.634	1.00	0.200	85.0000	90.3
Aldrin	13.949	1.00	0.200	68.0443	97.2
Azoxystrobin	29.64	1.00	0.200	90.9974	77.5
Bifenthrin	21.982	1.00	0.200	168.3717	79.5
Bromophos-methyl	14.613	1.00	0.200	71.9557	97.2
Bromopropylate	21.934	1.00	0.201	101.8729	90.0
Captan	15.57	2.01	0.402	86.8741	72.0
Carbophenothion	19.724	1.00	0.200	68.5728	96.2
Cyfluthrin	17.989	0.50	0.100	97.9695	74.4
Cypermethrin	11.115	1.00	0.200	57.2688	52.9
Chlorfenapyr	12.381	0.50	0.100	88.3344	75.1
Chlorothalonil	18.934	1.01	0.201	81.4200	78.8
Chlorpyrifos-methyl	26.141	2.01	0.401	83.8698	69.4
Chlorthiophos	26.454	1.00	0.200	94.5525	49.4
DDD 2,4	17.54	0.50	0.100	79.7596	38.1
DDE 4,4	17.326	0.50	0.100	84.6471	70.5
DDT 2,4	18.836	0.50	0.100	85.9344	72.3
Dicofol	14.388	1.00	0.200	88.0430	13.8
Dieldrin	17.413	1.00	0.200	334.0041	89.3
Endosulfan I	16.487	1.00	0.200	35.2980	41.6
Endosulfan II	18.223	1.00	0.200	98.9812	20.4
Endosulfan sulfate	19.86	1.00	0.200	92.7842	90.3
Endrin	18.115	1.00	0.201	75.4328	86.5
Fenarimol	24.021	1.00	0.200	101.3539	94.5
Fenitrothion	13.393	1.00	0.200	93.1230	94.4
Fenpropathrin	22.284	1.00	0.200	89.5164	73.1
Fenvalerate	28.047	1.00	0.200	91.5683	69.9
Folpet	15.766	2.00	0.400	69.8972	54.6
Phosalone	9.713	1.00	0.200	97.4683	35.9
Heptachlor	10.424	1.00	0.200	59.8488	39.1
Heptacloro epoxid	11.466	1.00	0.200	42.1311	32.3
Lambda cyhalothrin	10.628	1.00	0.200	135.7007	32.1
Methoxychlor	23.894	1.00	0.200	90.0305	93.5
Mirex	12.819	1.00	0.200	100.2361	87.6
Ovex (Clorfenson)	15.248	1.01	0.201	75.5841	90.9
Oxyfluorfen	22.119	1.00	0.200	72.0795	88.2
Parathion-methyl	23.62	0.50	0.100	55.6079	88.9
Permethrin	16.925	1.00	0.200	107.2255	93.1
Procymidone	17.667	2.00	0.400	86.3608	95.3
Profenofos	12.585	2.00	0.400	75.8056	96.7
Prothiofos	25.272	1.01	0.201	53.8811	42.1
Quintozene	23.024	1.00	0.200	0.0000	89.9
Tetradifon	15.688	1.00	0.200	69.3978	86.5
Trifluralin	17.228	2.00	0.401	138.5663	91.2
Vinclozolin	17.043	2.00	0.400	83.4945	94.7
HCH alpha	9.71	0.50	0.100	204.0544	*
HCH beta	22.81	2.00	0.401	1013.6074	76.4
HCH delta	9.148	1.00	0.200	73.8760	97.6
HCH gamma	12.517	1.00	0.200	79.4278	91.0

* Analytical error.

Initially, 1 µL of pesticide standards in acetonitrile-acetate was injected using a splitless liner, at an injector temperature of 250 °C, and carrier gas at a flow rate between 1.0 and 1.2 mL/min.

Four oven temperature ramp conditions were applied to determine the optimal conditions for better analytical sensitivity, as described below.

Condition 1: An initial column temperature of 80 °C, followed by a heating rate of 20 °C/min up to 160 °C, an increase to 255 °C at 5 °C/min, and a ramp of 20 °C/min to a final temperature of 280 °C, which was maintained for 1 min. The total runtime was 25.25 min.

Condition 2 (adapted from Maštovská et al.^43^): An initial column temperature of 80 °C, maintained for 1.5 min, followed by a 20 °C/min heating ramp up to 180 °C, an increase to 230 °C at 5 °C/min, and a ramp of 25 °C/min until a final temperature of 290 °C was reached, which was maintained for 10 min. The total runtime was 28.9 min.

Condition 3 (adapted from Faria et al.^44^): The column temperature ramp started at 60 °C, which was maintained for 1 min, followed by a heating rate of 30 °C/min up to 180 °C, an increase to 300 °C at 5 °C/min, and a ramp of 50 °C/min until a final temperature of 325 °C, which was maintained for 2 min. The total runtime was 29.5 min.

Condition 4 (adapted from Valenzuela et al.^45^): An initial column temperature of 60 °C, followed by a heating rate of 20 °C/min up to 160 °C, an increase to 255 °C at 5 °C/min, and a ramp of 20 °C/min to a final temperature of 280 °C, which was maintained for 7 min. The total runtime was 32.25 min.

Temperature ramps were optimized using injection volumes of 2, 5, and 8 µL. The evaluation of pesticide degradation in the injection system was conducted at injector temperatures of 100, 150, 200, and 250 °C.

### Method validation and greenness

The detection limit (DL) was calculated by multiplying the standard deviation (SD) by three^46^. The SD was obtained by assessing 10 white samples (extracts obtained from bat muscle only) and recording the abundance corresponding to the RT of each pesticide. One bat captured in PARNA Serra do Cipó was exclusively used to calculate the DL. The sample was from the reference area; therefore, high concentrations of pesticide residues were not expected. A larger bat was also chosen because it has more muscle tissue. Consequently, 10 extracts were prepared for the measurements and calculation of the SD. Little variation was expected in the values obtained because the samples were extracted from the same individual; the variations were attributed to the limitations of the instrument and extraction methods.

After determining the best extraction method, the recovery was calculated to observe the possible losses that occurred during the analytical process^47,48^. Two bat muscle fragments from a bat captured in PARNA Serra do Cipó were used. One fragment was fortified with a pesticide stock solution of standards containing 69 pesticides before extraction and the other was fortified after extraction. Thereafter, both fragments were subjected to chromatographic runs to determine the analytes and the estimated recovery values. The recovery indicates the amount of analyte detected in relation to the amount added to the sample. Variations in the values may occur because of matrix effects and loss of analytes due to degradation in the injection system or extraction procedure (cleanup, dilution, drying, or pre-concentration).

The greenness of the developed method was determined using Green Analytical Procedure Index (GAPI)^49^ and Analytical EcoScale (AES)^50^ metric systems.

### Ethical approval

The study was conducted according to the Declaration of Helsinki and ARRIVE guidelines, and approved by the Ethics Committee on the Use of Animals at the Federal University of Minas Gerais (Protocol CEUA 166/2017) and by the Chico Mendes Institute for Conservation and Biodiversity (Protocol ICMBio 57,026-1). the study is reported in accordance with.

### Consent to participate

All the authors agreed to participate in the publication.

## Results

**Figure 1:**
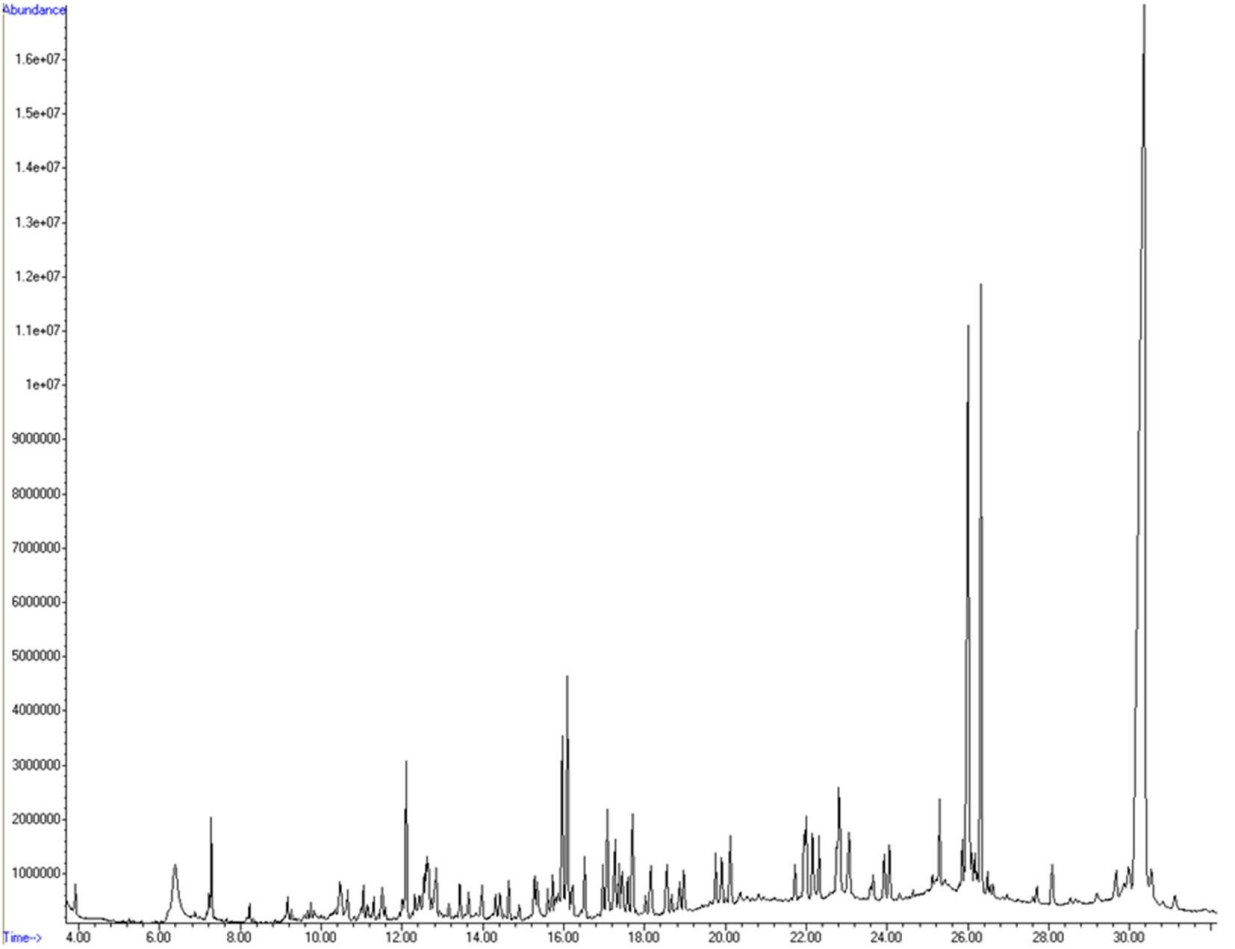
Chromatogram of blank bat muscle sample spiked with 69 pesticides obtained via gas chromatography coupled with mass spectrometry (GC–MS) in full-scan mode using Condition 4 (initial column temperature of 60 °C, followed by a heating rate of 20 °C/min up to 160 °C, an increase to 255 °C at 5 °C/min, and a ramp of 20 °C/min to a final temperature of 280 °C, which was maintained for 7 min; the total runtime was 32.25 min).

The miniaturized QuEChERS method (Method B) presented the optimal results for the extraction as it produced discernible peaks and less noise in the spectra. Subsequently, the sample extraction, cleanup method, and chromatographic conditions were optimized. Four oven temperature ramps (Conditions 1–4) were tested, and Conditions 1 and 4 showed the best results. These conditions were tested again with an injection volume of 2 µL. Condition 4 was chosen because it had less noise and a better peak definition (Fig. 1). The chosen method was then tested using injection volumes of 5 and 8 µL. An injection volume of 8 µL resulted in the detection of a greater number of pesticides.

To determine whether pesticide degradation occurred in the injection system, injector temperatures of 100, 150, 200, and 250 °C were also tested.

For data acquisition, three ion transitions were detected for each pesticide at their respective RTs using this method. The pesticides were identified and confirmed by comparing the mass spectra obtained in the full-scan mode with the NIST library^51^. A minimum probability of 70% was applied between the spectrum obtained in full-scan mode and the library database to confirm the identification of the analyte. This percentage was considered adequate because the tests were performed using analytical standards. Differences in probability were obtained by comparing the spectra obtained in the full-scan and SIM modes. These differences occur because it is possible to view all the ions present in full-scan mode, whereas only the selected ions are displayed in SIM mode. In the SIM spectrum, the analyte was quantified by estimating the corresponding peak area. The DL estimations are listed in Table 3.

**Table 3:** Retention time (RT), recovery, standard deviation (SD), and detection limit (DL) of the compounds analyzed via gas chromatography coupled with mass spectrometry (GC–MS).

Compound	RT	%	SD	DL
Alachlor	12.63	90.3	42,487.12	127,461
Aldrin	13.95	97.2	150,304.90	450,915
Azoxystrobin	29.64	77.5	9942.20	29,827
Bifenthrin	21.98	79.5	5078.33	15,235
Bromophos-methyl	14.61	97.2	24,018.28	72,055
Bromopropylate	21.93	90	4565.86	13,698
Captan	15.57	72	34,961.06	104,883
Carbophenothion	19.72	96.2	30,464.97	91,395
Chlorfenapyr	17.99	74.4	9395.30	28,186
Chlorothalonil	11.16	52.9	9080.17	27,241
Chlorpyrifos	18.93	78.8	23,983.91	71,952
Chlorpyrifos-methyl	12.38	75.1	56,800.25	170,401
Cyfluthrin	26.14	69.4	20,108.86	60,327
Cyhalothrin-lambda	23.89	93.5	4945.55	14,837
Cypermethrin	26.45	49.4	20,336.56	61,010
DDD 2,4	17.54	38.1	3669.19	11,008
DDE 4,4	17.33	70.5	3315.94	9948
DDT 2,4	18.84	72.3	2612.30	7837
Dicofol	14.39	13.8	30,104.87	90,315
Dieldrin	17.41	89.3	1647.39	4942
Endosulfan I	16.49	41.6	8405.06	25,215
Endosulfan II	18.22	20.4	29,272.27	87,817
Endosulfan sulfate	19.86	90.3	28,347.78	85,043
Endrin	18.12	86.5	27,846.99	83,541
Fenarimol	24.02	94.5	6130.06	18,390
Fenitrothion	13.39	94.4	21,292.26	63,877
Fenpropathrin	22.28	73.1	43,522.44	130,567
Fenvalerate alpha	28.057	69.9	17,002.46	51,007
Folpet	15.77	54.6	61,354.62	184,064
HCH alpha	9.71	35.9	1867.91	5604
HCH beta	10.42	39.1	16,943.89	50,832
HCH delta	11.47	32.3	8203.15	24,609
Heptachlor	12.82	87.6	8458.18	25,375
Heptacloro epoxid	15.25	90.9	14,973.71	44,921
Hexachlorobenzene	10.63	32.1	4154.33	12,463
Methoxychlor	22.12	88.2	9494.075	28,482
Mirex	23.62	88.9	10,877.95	32,634
Ovex (Clorfenson)	16.93	93.1	18,757.17	56,272
Oxyfluorfen	17.67	95.3	3331.79	9995
Parathion-methyl	12.59	96.7	56,303.56	168,911
Permethrin	25.27	42.1	53,311.78	159,935
Phosalone	23.02	89.9	169,183.28	507,550
Procymidone	15.69	86.5	71,457.31	214,372
Profenofos	17.23	91.2	16,225.92	48,678
Prothiofos	17.04	94.7	28,589.13	85,767
Tetradifon	22.81	76.4	981,945.32	2,945,836
Trifluralin	9.15	97.6	467.27	1402
Vinclozolin	12.52	91	28,995.68	86,987

The recovery values ranged from 35.3 to 97.6%. According to the Association of Official Analytical Chemists^48^, the recommended range of recovery percentages for analytes at a concentration of 1 ppb varies from 40 to 120%^48^. Seven pesticides (trifluralin, HCH alpha, HCH beta, endosulfan I, dieldrin, bifenthrin, and lambda-cyhalothrin) showed recovery values outside the recommended range (Table 3). However, as the NIST library was used as a confirmatory method, only endosulfan I and lambda-cyhalothrin did not show acceptable recovery. Therefore, the extraction method we developed yielded satisfactory results.

**Figure 2:**
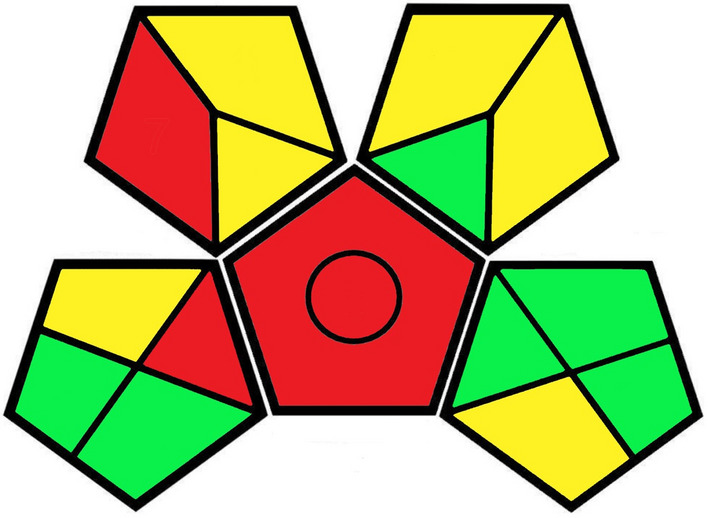
Green Analytical Procedure Index **(**GAPI) evaluation pictogram of the developed analytical method.

The developed method was evaluated for greenness using GAPI and AES. The estimation parameters of the GAPI are presented in Table 4 and a pictogram is shown in Fig. 2. For the greenness evaluation using AES, the method obtained a score of 80 (Table 5), which indicates an excellent green analysis.

**Table 4:** Green Analytical Procedure Index estimation of the developed analytical method.

Category	Criteria	Color
**I Sample preparation**		
1 Collection	Offline	Red
2 Preservation	None	Green
3 Transport	None	Green
4 Storage	Under normal condition	Yellow
5 Type of method	Extraction required	Red
6 Scale of extraction	Microextraction	Yellow
7 Solvents/reagents used	Non-green reagents used	Red
8 Additional treatments	Simple treatments	Yellow
**II Reagent and solvents**		
9 Amount	< 10 mL (< 10 g)	Green
10 Health hazard	NFPA health hazard scores: Acetone-2; Acetonitrile-3; Hexane-1	Yellow
11 Safety hazard	NFPA Flammability scores: Acetone-3; Acetonitrile-3; Hexane-3	Yellow
**III Instrumentation**		
12 Energy	≤ 0.1 kWh per sample	Green
13 Occupational hazard	Hermetic sealing of analytical process	Green
14 Waste	< 1 mL (1 g)	Green
15 Waste treatment	Degradation	Yellow

**Table 5:** Analytical EcoScale score points of the developed analytical method.

Category	Criteria	Penalty Points (PP)
Reagents	Acetonitrile (< 10 mL/sample)	4
	Hexane (< 10 mL/sample)	8
	Acetone (< 10 mL/sample)	4
Instrument energy	GC/MS (> 1.5 kWh/sample)	2
Occupational hazard	Hermetization of analytical process	0
Waste	< 1 mL (< 1 g)	1
	Degradation	1
Total PP		20
AES score	100-PP	80

No residual pesticides were detected above the DLs in the muscle tissues of bats from Uberaba and PARNA Serra do Cipó. Similarly, no residual pesticides were detected in the extracts obtained from the liver and adipose tissues.

## Discussion

In this study, we developed a method for determining the residue of 48 pesticides in bat muscle using GC–MS. A miniaturized QuEChERS method adapted from Brandhonneur et al.^25^ presented optimal results as it yielded discernible peaks and less baseline noise. Miniaturization of the method makes analysis feasible even when the sample quantity is limited. In addition, it uses fewer reagents than traditional methods, reducing both the cost and impact on the environment and health of researchers.

Acetonitrile is one of the most commonly used extraction solvents because it allows the extraction of many pesticides while minimizing the extraction of lipids, carbohydrates, and proteins that are present in the matrix^52^. Lipids are compounds that warrant more attention because they can compromise the quality of results and can also be deposited in the injection system or chromatographic column, damaging the chromatographic system^53^. The hexane added to the extraction process assists in the removal of lipophilic compounds because these compounds are less soluble in acetonitrile^54^. Drying salts, such as magnesium sulfate (MgSO_4_) and sodium sulfate (Na_2_SO_4_), remove residual water from the solution and facilitate the removal of polar components from the matrix^14,52,55^. In this work, we used MgSO_4_ because it has greater drying power than Na_2_SO_4_^52^. In addition, the heat released during the chemical hydration reaction of MgSO_4_ can contribute to pesticide extraction^14^.

Furthermore, we used PSA and C18 sorbents to remove co-extracted interferents from the matrix^14,56,57^ during sample cleanup. PSA has a bidentate structure that exerts a chelating effect, which enables the retention of free fatty acids, carbohydrates, and other polar compounds present in the matrix^14^, whereas C18 is important for the removal of fatty acids and other non-polar components^56^.

According to the validation guide for quality control methods and procedures for the analysis of pesticide residues, for the analysis by CG-MS with a simple quadrupole mass analyzer to be valid, the data must be acquired in full-scan method, with a limited range of m/z and SIM mode monitoring of three ions^46^. In the full-scan mode, a complete mass scan was performed in the range of 50–450 m/z, generating a full spectrum that contained more than one substance at the same RT. This data acquisition mode is less sensitive when analytes are present at low concentrations, whereas high concentrations of matrix interferents are present^58,59^. The sensitivity and selectivity of the method can be improved using SIM mode, in which the mass analyzer is programmed to monitor only the characteristic ions of the studied compounds^59^.

The developed method allowed for the detection of 48 pesticides using GC–MS. Other methods have been used to detect pesticides in bats^32,33^. Valdespino and Sosa^33^ also identified 19 organochlorine pesticides using GC–MS. Stecherts et al.^32^ analyzed 25 organochlorine, organophosphate, and pyrethroid pesticides in bat carcasses using three different chromatographic systems (GC/ECD, HPLC/DAD, and LC/MS/MS). Thus, the method described in this study allows for the detection of a greater number of pesticides. Furthermore, both aforementioned methods required the use of the whole bat carcass, whereas our method used only 250 mg of bat muscle, allowing the use of the rest of the animal for other analyses, which presents a great advantage for future studies of environmental toxicology.

Previous studies evaluated the exposure of insectivorous bats by determining the residues of organochlorine and organophosphate insecticides^2^. However, no residual pesticide was detected above the DLs in bats from either Uberaba or PARNA Serra do Cipó. PARNA Serra do Cipó is an integral protection conservation unit that is not surrounded by intensive agricultural activities^36^. In contrast, Uberaba is one of the main municipalities in the state of Minas Gerais that produces grains and sugarcane^35^, and the use of pesticides for these crops is higher than that for other crops in Brazil^60^. Literature on environmental contamination by pesticides in these municipalities is scarce. However, analyses of the water supply to city inhabitants have revealed contamination by alachlor, atrazine, carbendazim, chlordane, DDT, DDD, DDE, diuron, glyphosate, lindane, mancozeb, permethrin, trifluralin, 2,4-D, 2,4, 5-T, aldicarb, aldrin, carbofuran, chlorpyrifos, endosulfan, endrin, methamidophos, metalachlor, molinate, methyl parathion, pendimenthalin, profenofos, simazine, tebuconazole, and terbufos^61^. Therefore, although pesticide residues were not detected, it is reasonable to assume that bats in Uberaba are exposed to environmental contamination by pesticides, with concentrations below those defined in the DLs.

The greenness of the developed analytical method was estimated using two metric systems: GAPI^49^ and AES^50^. GAPI is a qualitative analysis that measures 15 parameters that are divided into three categories: I, sample preparation (collection, preservation, transport, storage, type of method, scale of extraction, solvents/reagents used, and additional treatments); II, reagents and solvents (amount, health hazard, and safety hazard); and III, instrumentation assessment (energy consumption, occupational hazard, waste produced, and waste treatment). Each parameter is color coded according to the estimated environmental impact as follows: low (green), medium (yellow), or high (red); and the results are presented as a pictogram formed by five pentagons^49,62^. The GAPI pictogram for the method described herein exhibited a lower estimated environmental impact than those of previous QuEChERS methods^63^.

In this study, we used the AES metric system^50^ to evaluate the greenness of the developed method. AES is based on EcoScale, a semi-quantitative analysis for measuring the ecological, safety, and economic impacts of organic synthesis methods^64^. AES attribute scores for the analytical method range from 0 to 100. Penalty points are calculated based on reagent amounts and hazards, energy consumption, occupational hazards, and waste, which are then subtracted from the maximum score of 100. Excellent green analytical methods have scores higher than 75, and scores higher than 50 are considered acceptable^50,62^. The method described in this study obtained a score of 80, which indicates an excellent green analysis.

In summary, the analytical method used in this study allowed the identification of 48 different pesticides present in bat muscle using GC–MS. However, no pesticide residues were detected in the 148 analyzed bats from the two different areas.

## Data Availability

The datasets generated during and/or analysed during the current study are available from the corresponding author on reasonable request.

## References

[1] Köhler HR, Triebskorn R (2013). Wildlife ecotoxicology of pesticides: can we track effects to the population level and beyond?. Science.

[2] Torquetti CG, Guimarães ATB, Soto-Blanco B (2021). Exposure to pesticides in bats. Sci. Total Environ..

[3] Geiger F (2010). Persistent negative effects of pesticides on biodiversity and biological control potential on European farmland. Basic Appl. Ecol..

[4] Beketov MA, Kefford BJ, Schäfer RB, Liess M (2013). Pesticides reduce regional biodiversity of stream invertebrates. Proc. Natl. Acad. Sci. USA.

[5] Clark DR, Bagley FM, Johnson WW (1988). Northern Alabama colonies of the endangered grey bat *Myotis grisescens*: organochlorine contamination and mortality. Biol. Conserv..

[6] Mitchell-Jones AJ, Cooke AS, Boyd IL, Stebbings RE (1989). Bats and remedial timber treatment chemicals – a review. Mamm. Rev..

[7] Clark DR (2001). DDT and the decline of free-tailed bats (*Tadarida brasiliensis*) at Carlsbad Cavern, New Mexico. Arch. Environ. Contam. Toxicol..

[8] Mineau P, Whiteside M (2013). Pesticide acute toxicity is a better correlate of U.S. grassland bird declines than agricultural intensification. PloS One.

[9] Sparling DW, Fellers GM, McConnell LL (2001). Pesticides and amphibian population declines in California, USA. Environ. Toxicol. Chem..

[10] Davidson C (2004). Declining downwind: amphibian population declines in California and historical pesticides use. Ecol. Appl..

[11] Alder L, Greulich K, Kempe G, Vieth B (2006). Residues analysis of 500 high priority pesticides: better by GC-MS or LC-MS/MS?. Mass Spectrom. Rev..

[12] Lehotay SJ (2008). Identification and confirmation of chemical residues in food by chromatography-mass spectrometry and other techniques. Trends Anal. Chem..

[13] Hercegová A, Dömötörová M, Matisová E (2007). Sample preparation methods in the analysis of pesticide residues in baby food with subsequent chromatographic determination. J. Chromatogr. A.

[14] Anastassiades M, Lehotay SJ, Stajnbaher D, Schenck FJ (2003). Fast and easy multiresidue method employing acetonitrile extraction/partitioning and "dispersive solid-phase extraction" for the determination of pesticide residues in produce. J. AOAC Int..

[15] Oliveira FA, Reis LPG, Soto-Blanco B, Melo MM (2015). Pesticides residues in the *Prochilodus costatus* (Valenciennes, 1850) fish caught in the São Francisco River, Brazil. J. Environ. Sci. Health B.

[16] Oliveira FADS, Pereira ENC, Gobbi JM, Soto-Blanco B, Melo MM (2018). Multiresidue method for detection of pesticides in beef meat using liquid chromatography coupled to mass spectrometry detection (LC-MS) after QuEChERS extraction. Food Addit. Contam. Part A Chem. Anal. Control Expo. Risk Assess..

[17] Oliveira FAS (2014). Optimization of chromatographic conditions and comparison of extraction efficiencies of four different methods for determination and quantification of pesticide content in bovine milk by UFLC-MS/MS. Quím. Nova.

[18] Tette PA (2016). Multiclass method for pesticides quantification in honey by means of modified QuEChERS and UHPLC-MS/MS. Food Chem..

[19] Almeida MO (2020). Optimization of method for pesticide detection in honey by using liquid and gas chromatography coupled with mass spectrometric detection. Foods.

[20] Pinheiro CGMDE, Oliveira FAS, Oloris SCS, Silva JBA, Soto-Blanco B (2020). Pesticide residues in honey from the stingless bee *Melipona subnitida* (Meliponini, Apidae). J. Apic. Sci..

[21] Brondi SHG, Macedo AN, Vicente GHL, Nogueira ARA (2011). Evaluation of the QuEChERS method and gas chromatography-mass spectrometry for the analysis pesticides residues in water and sediment. Bull. Environ. Contam. Toxicol..

[22] Cerqueira MBR (2014). Evaluation of the QuEChERS method for the extraction of pharmaceuticals and personal care products from drinking-water treatment sludge with determination by UPLC-ESI-MS/MS. Chemosphere.

[23] Asenio-Ramos M, Hernández-Borges J, Ravelo-Pérez LM, Rodríguez-Delgado MA (2010). Evaluation of a modified QuEChERS method for the extraction of pesticides from agricultural, ornamental and forestal soils. Anal. Bioanal. Chem..

[24] Bragança I, Plácido A, Paíga P, Domingues VF, Delerue-Matos C (2012). QuEChERS: a new sample preparation approach for the determination of ibuprofen and its metabolites in soils. Sci. Total Environ..

[25] Brandhonneur N (2015). A micro-QuEChERS method coupled to GC-MS for the quantification of pesticides in specific maternal and fetal tissues. J. Pharm. Biomed. Anal..

[26] Stöckelhuber M (2017). Determination of pesticides adsorbed on arthropods and gastropods by a Micro-QuEChERS approach and GC-MS/MS. Chromatographia.

[27] Jesús F (2018). Miniaturized QuEChERS based methodology for multiresidue determination of pesticides in odonate nymphs as ecosystem biomonitors. Talanta.

[28] Gałuszka A, Migaszewski A, Namieśnik J (2013). The 12 principles of green analytical chemistry and the SIGNIFICANCE mnemonic of green analytical practices. Trends Anal. Chem..

[29] Kunz TH, Torrez EB, Bauer D, Lobova T, Fleming TH (2011). Ecosystem services provided by bats. Ann. N. Y. Acad. Sci..

[30] Benton AH (1951). Effects on Wildlife of DDT used for control of Dutch Elm disease. J. Wildl. Manag..

[31] Dalquest, W. W. Mammals of the Mexican state of San Luis Potosi. Doctoral Thesis, Louisiana State University and Agricultural & Mechanical College (1951). https://digitalcommons.lsu.edu/gradschool_disstheses/7990.

[32] Stecherts C, Kolb M, Bahadir M, Djossa BA, Fahr J (2014). Insecticide residues in bats along a land use-gradient dominated by cotton cultivation in northern Benin, West Africa. Environ. Sci. Pollut. Res. Int..

[33] Valdespino C, Sosa VJ (2017). Effect of landscape tree cover, sex and season on the bioaccumulation of persistent organochlorine pesticides in fruit bats of riparian corridors in eastern Mexico. Chemosphere.

[34] Altringham JD (2011). Bats, from evolution to conservation.

[35] SEAPA (Secretaria do Estado de Agricultura, Pecuária e Abastecimento) *Projeções do Agronegócio – Minas Gerais – 2017–2027*, 3rd ed. (SEAPA, 2018).

[36] ICMBio (Instituto Chico Mendes de Conservação da Biodiversidade) *Plano de Manejo do Parque Nacional da Serra do Cipó e Área de Proteção Ambiental Morro da Pedreira* (ICMBio, 2009).

[37] Sikes RS, Animal Care and Use Committee of the American Society of Mammalogists (2016). Guidelines of the American Society of Mammalogists for the use of wild mammals in research and education. J. Mammal.

[38] Jefferies DJ (1972). Organochlorine inseticide residues in British bats and their significance. J. Zool..

[39] Covaci A, Gheorghe A, Schepens P (2004). Distribution of organochlorine pesticides, polychlorinated biphenyls and α-HCH enantiomers in pork tissues. Chemosphere.

[40] Barbieri MV (2019). Analysis of 52 pesticides in fresh fish muscle by QuEChERS extraction followed by LC-MS/MS determination. Sci. Total Environ..

[41] Freitas MB, Welker AF, Pinheiro EC (2006). Seasonal variation and food deprivation in common vampire bats (Chiroptera: Phyllostomidae). Braz. J. Biol..

[42] Codex Alimentarius. *Guidelines on performance criteria for methods of analysis for the determination of pesticide residues in food and feed*. CXG90-2017 (FAO, 2017).

[43] Maštovská K, Lehotay SJ, Anastassiades M (2005). Combination of analyte protectants to overcome matrix effects in routine GC analysis of pesticide residues in food matrixes. Anal. Chem..

[44] Faria, V. H. F. *et al*. Avaliação de resíduos de agrotóxicos em polpas de morango industrializadas. *Pesticidas Revista de Ecotoxicologia e Meio Ambiente* **19**, 49-56 (2009).

[45] Valenzuela EF, Menezes HC, Cardeal ZL (2019). New passive sampling device for effective monitoring of pesticides in water. Anal. Chim. Acta.

[46] EC (European Commission). *Guidance document on analytical quality control and method validation procedures for pesticides residues analysis in food and feed*. SANTE/12682/2019 (DG SANTE, 2019).

[47] Brasil – Ministério da Agricultura, Pecuária e Abastecimento. Secretaria de Defesa Agropecuária. *Guia de validação e controle de qualidade analítica: fármacos em produtos para alimentação e medicamentos veterinários* (Ministério da Agricultura, Pecuária e Abastecimento, 2011).

[48] AOAC International. *Appendix F: Guidelines for Standard Method Performance Requirements*. AOAC Official Methods of Analysis (AOAC International, 2016).

[49] Płotka-Wasylka J (2018). A new tool for the evaluation of the analytical procedure: green analytical procedure index. Talanta.

[50] Gałuszka A, Migaszewski ZM, Konieczka P, Namieśnik J (2012). Analytical Eco-Scale for assessing the greenness of analytical procedures. Trends Anal. Chem..

[51] Tahboub YR, Zaater MF, Al-Talla ZA (2005). Determination of the limits of identification and quantitation of selected organochlorine and organophosphorous pesticide residues in surface water by full-scan gas chromatography/mass spectrometry. J. Chromatogr. A.

[52] Lehotay S, Lightfield AR, Harman-Fetcho JA, Donoghue DJ (2001). Analysis of pesticides residues in eggs by direct sample introduction/gas chromatography/tandem mass spectrometry. J. Agric. Food Chem..

[53] Hajšlová J, Zrostlíková J (2003). Matrix effects in (ultra)trace analysis of pesticide residues in food and biotic matrices. J. Chromatogr. A.

[54] Przybylski C, Segard C (2009). Method for routine screening of pesticides and metabolites in meat based baby-food using extraction and gas chromatography-mass spectrometry. J. Sep. Sci..

[55] Lehotay S (2000). Analysis of pesticide residues in mixed fruit and vegetable extracts by direct sample introduction/gas chromatography/tandem mass spectrometry. J. AOAC Int..

[56] Georgakopoulos P (2001). Optimization of octadecyl (C18) sorbent amount in QuEChERS analytical method for the accurate organophosphorus pesticide residues determination in low-fatty baby foods with response surface methodology. Food Chem..

[57] Lehotay SJ (2010). Comparison of QuEChERS sample preparation methods for the analysis of pesticides residues in fruits and vegetables. J. Chromatogr. A.

[58] Peña F, Cárdenas S, Gallego M, Calcárcel M (2002). Analysis of phenylrea herbicides from plants by GC/MS. Talanta.

[59] Soboleva E, Ahad K, Ambrus A (2004). Applicability of some mass spectrometric criteria for the confirmation of pesticide residues. Analyst.

[60] Bombardi, L. M. *Geografia do Uso de Agrotóxicos no Brasil e Conexões com a União Europeia* (FFLCH-USP, 2017).

[61] Brasil – Ministério da Saúde. Secretaria de Vigilância em Saúde. Departamento de Saúde Ambiental, do Trabalhador e Vigilância das Emergências em Saúde Pública. *Indicadores institucionais do Programa Nacional de Vigilância da Qualidade da Água para consumo humano – 2019* (Ministério da Saúde, 2020).

[62] Sajid M, Płotka-Wasylka J (2022). Green analytical chemistry metrics: a review. Talanta.

[63] Billiard KM, Dershem AR, Gionfriddo E (2020). Implementing green analytical methodologies using solid-phase microextraction, a review. Molecules.

[64] Van Aken K, Strekowski L, Patiny L (2006). EcoScale, a semi-quantitative tool to select an organic preparation based on economical and ecological parameters. Beilstein J. Org. Chem..

